# Disease of the Antrum Produced by a Fall

**Published:** 1853-07

**Authors:** 


### ARTICLE XII.

### *Disease of the Antrum produced by a Fall.*

MR. LEVISON reports the following case in the *London Lancet.* Some time since, a young woman of this place, about twenty-two years old, applied for my advice, she having a large swelling on the right cheek, the size of a turkey's egg, the lower or broadest part of the tumor being in a line with the upper lip; the swelling extending so high as to affect the eye and eyelid. The former was protruded, and the latter almost paralyzed, so that, besides the deformity, the sight of the right eye was seriously affected, and the secretion of tears a source of irritation to the cheek. The tumor was very hard; the surface of the skin, red, inflamed, shining, and very painful to the touch.

Considering that disease was connected with the pituitary membrane which lines the antrum, I carefully examined the mouth, and observed that the second molar tooth on the affected side was carious, and the gum dark and livid, and so soft, that it appeared to have been deprived of all vitality; and at the same time, the fetor was very offensive. In fact, the gum presented a similar appearance as when there is an effort to throw a piece of dead bone. In the present case there was a well-defined line of demarcation between the affected portion (the cause of the local irritation) and the healthy jaw, for the shape of the diseased gum corresponded to a piece of bone I could move with the slightest pressure. There had not been any teeth extracted on the diseased side of the face, and it occurred to me that the affection of the antrum had been induced by a blow on the face, or from a fall. That, in either case, a large portion of the alveolar process had been fractured, and the molar tooth injured at the same time, by having its periosteum denuded, or so injured that the inflammatory action was set up, and the tooth ultimately destroyed. Having questioned my patient, her answers proved the correctness of my diagnosis. She told me that about ten years since she had fallen down stairs on her face, but that she was not aware that she injured herself at the time, having been stunned. She distinctly recollected that, soon after the accident, the swelling on her face commenced with some uneasiness in her mouth; that at first the tumor was very small, and when it had attained the size of a pigeon's egg, she had applied for advice, and was given something to rub it externally, but without any advantage; and that it had since gone on increasing; that she did not then heed it much, although it was always more or less painful; but that she never suffered any alarm until there seemed every probability she should lose her sight. I mention these particulars because few persons of her class give any history of their cases. It can only be obtained by a species of cross-examination.

I removed the carious molar tooth, with the portion of the dead alveolar process, when a considerable quantity of thick, curdy matter came away, some portions being in different sized lumps. A direct communication was kept up between the antrum and the mouth by means of a conical-shaped tube, and the discharge continued, night and day, for some time. For three weeks I injected the antrum, every alternate day, with a lotion of about twenty drops of the chloride of zinc to an ounce of distilled water. About the end of a month the deformity had entirely disappeared, and the affected side had acquired its normal proportions; the eye had recovered its natural position and brightness, and the mouth itself had become perfectly healthy.

